# Acute drug intoxication: predictive factors for hospitalization

**DOI:** 10.11604/pamj.2025.52.23.47923

**Published:** 2025-09-18

**Authors:** Fatma Medhioub Kaaniche, Farah Zouari, Salma Jerbi, Ines Dahech, Yosr Ben Taher, Arij Abdellatif, Wiem Feki, Zina Hakim, Dorsaf Dlensi, Rania Allala

**Affiliations:** 1 Intensive Care Department, Mahres Regional Hospital, Faculty of Medicine of Sfax, University of Sfax, Sfax, Tunisia,; 2 Radiology Department, Hedi Chaker Hospital, Faculty of Medicine of Sfax, University of Sfax, Sfax, Tunisia,; 3 Occupational Medicine Unit, Mahres Regional Hospital, Faculty of Medicine of Sfax, University of Sfax, Sfax, Tunisia

**Keywords:** Intoxication, medications, emergency, hospitalization

## Abstract

**Introduction:**

acute drug intoxication (ADI) is a common reason for emergency department visits and intensive care unit admissions. The objective of our study was to describe the population presenting to the emergency department for ADP and to identify predictive factors for hospitalization.

**Methods:**

this was a cross-sectional study conducted over a 30-month period (July 1, 2021 - December 31, 2023). We included all patients admitted with a diagnosis of acute drug poisoning.

**Results:**

a total of 41 cases of ADP were recorded. Independent predictive factors for hospitalization included: poly-drug poisoning (OR = 3.3; 95% CI [2.6-6.7]; p = 0.031), toxic dose exposure (OR = 1.7; 95% CI [1.1-3.5]; p = 0.04), paracetamol poisoning (OR = 1.4; 95% CI [1.2-2.7]; p = 0.02), oral antidiabetic drug poisoning (OR = 1.1; 95% CI [1.1-2.8]; p = 0.01), benzodiazepine poisoning (OR = 1.9; 95% CI [0.8-3.2]; p = 0.02), miosis (OR = 1.2; 95% CI [1.2-4.2]; p = 0.043), seizures (OR = 1.3; 95% CI [0.8-2.1]; p = 0.001), sinus bradycardia (OR = 1.3; 95% CI [0.7-2.7]; p = 0.031), and conduction disturbances (OR = 1.8; 95% CI [1.4-4.2]; p = 0.02).

**Conclusion:**

these findings highlight the substantial number of potentially avoidable hospitalizations. Developing a specific guideline for drug poisoning could help standardize management in emergency settings.

## Introduction

Acute drug intoxications (ADIs), defined as toxic exposures to one or more pharmaceutical substances, represent a major and growing global public health concern. They are among the most common causes of consultation in emergency departments and admissions to intensive care units worldwide [1]. ADIs can result from intentional ingestions, such as suicide attempts, or from accidental exposures, and they affect all age groups and social categories. The burden of ADIs is not limited to their high incidence or associated mortality; it also lies in the potential severity of their clinical consequences. The prognosis often depends on several critical factors, including the type and dose of the substance ingested, the time elapsed before medical intervention, and the quality and promptness of emergency care. In many cases, rapid and appropriate management can significantly improve outcomes, while delays in diagnosis or treatment may lead to life-threatening complications. In low- and middle-income countries, such as Tunisia, emergency departments frequently face the challenge of managing diverse and complex ADI cases, often with limited toxicological resources. Understanding the patterns and predictors of severity in these intoxications is essential for optimizing triage, improving patient outcomes, and guiding public health interventions. The primary objectives of this study are twofold: first, to describe the epidemiological, clinical, biological, and therapeutic characteristics of patients presenting with acute drug intoxication at the emergency department of Mahres Regional Hospital; and second, to identify the predictive factors associated with hospitalization in this patient population.

## Methods

**Study design and setting:** we conducted a cross-sectional observational study in the Emergency Department of Mahres Regional Hospital, Tunisia, over a 30-month period (July 1, 2021-December 31, 2023).

**Eligibility criteria and participants:** all patients admitted with acute drug intoxication (ADI), identified via self-report, family report, or clinical/toxicological evaluation, were eligible. Both intentional (e.g., suicide attempts) and accidental ingestions were included. Patients with incomplete data, unconfirmed intoxications, non-drug-related poisonings (e.g., alcohol, household chemicals), or cases immediately fatal before assessment were excluded.

**Sampling strategy and sample size:** a consecutive non-probabilistic sampling method was used; all eligible patients during the study period were enrolled. The sample size corresponded to the total number of eligible cases, providing a representative overview of ADI cases in this hospital setting.

**Data collection:** data were collected at admission using a standardized clinical record form completed by attending physicians and verified by the principal investigator. Recorded variables included demographic and epidemiological characteristics, details of the intoxication (substance(s), dose, intent), clinical presentation, laboratory and toxicology results, therapeutic interventions, and immediate outcomes. All data were anonymized and entered into a secure digital database.

**Outcome measures:** the primary outcome was hospitalization following ADI. Patients requiring hospitalization (H+ group) were compared with those managed without admission (H- group) to identify predictive factors.

**Statistical analysis:** analyses were performed using SPSS version 25. Descriptive statistics summarized patient characteristics, with categorical variables reported as counts and percentages and continuous variables as means ± standard deviations or medians with interquartile ranges. Comparisons between H+ and H- groups used Chi-square or Fisher's exact tests for categorical variables and Student's t-test or Mann-Whitney U test for continuous variables, as appropriate. Variables showing potential associations in univariate analysis were entered into a multivariate logistic regression model to determine independent predictors of hospitalization, with adjusted odds ratios (ORs) and 95% confidence intervals (CIs) reported. Model fit was assessed using the Hosmer-Lemeshow test, and multicollinearity was checked via variance inflation factors (VIFs). A p-value < 0.05 was considered statistically significant. All analyses were detailed to ensure reproducibility by other researchers.

**Ethical approval:** the study was conducted in accordance with the principles of the Declaration of Helsinki. Patient confidentiality was maintained throughout the research process, and the study protocol received ethical approval from the institutional review board of Mahres Regional Hospital.

## Results

### The general characteristics of the study population

During the 30-month study period, 35,723 patients presented to the Emergency Department of Mahres Regional Hospital, among whom 41 cases of acute drug intoxication (ADI) were identified, representing 0.11% of all admissions and 61.2% of acute poisonings. Patients had a mean age of 21.6 ± 9 years (range: 5-71), with predominance of young adults aged 15-25 years (58.5%) and women (sex ratio M/F = 0.28). Most patients were from rural areas (82.9%), and suicidal self-poisoning was the main circumstance (87.8%), often related to family problems (39%). Medical or surgical comorbidities were present in 22%, toxic habits in 12.2%, prior psychiatric follow-up in 19.5%, and previous intoxications in 14.6%. The median delay before hospital admission was 2 hours, with 70.7% presenting within 4 hours. Intoxications were multi-drug in 53.7%, and the most frequent substances were paracetamol (65.9%) and proton pump inhibitors (46.3%) (Table 1).

**Table 1:** distribution of acute drug poisoning cases by pharmacological class and specific agents among patients at Mahres Regional Hospital, Tunisia, July 2021 - December 2023 (N = 41)

Pharmacological class	Molecule	Effective	Frequency % (n=41)
Paracetamol	-Level 1: Doliprane Adol Cetamol -Level 2: Co-algesic	27	65.9%
PPI	-Mopral -Esomeprazole -Ippsium	19	46.3%
NSAIDs	-Piroxen -Diclofen -Celebrex -Apranax	17	41.5%
Oral antidiabetic drugs (Biguanides)	-Glucophage -Metformin	4	9.4%
Antihypertensives	-IEC: Lopril Tensopril -IC: Tildiem -BB: Cincor	14	34.1%
Antibiotics	-Amoxicillin: Clamoxyl -Amox-Ac.Clav: Augmentin -C3G: Oroken -Quinolones: Cipro	11	26.8%
Tricyclic antidepressants	-Anafranil -Laroxyl -Tofranil -Prozac	10	24.4%
Anxiolytics (Benzodiazepine)	-Lexomil -Lysanxia -Seresta	16	39%
Neuroleptics	-Haldol -Risperidone	5	12.2%
Anti-vertigo	-Tanganil	1	2.4%

Clinical presentation was diverse (Table 2), and laboratory investigations revealed hypoglycemia (7.3%), renal impairment (4.9%), hepatic cytolysis (14.6%), rhabdomyolysis (2.4%), metabolic acidosis (12.2%), and respiratory alkalosis (2.4%). Chest X-rays were abnormal in 2 patients (pneumonitis), and ECG abnormalities, particularly sinus bradycardia and conduction disorders, were noted in 29.3% of cases. Therapeutic interventions included oxygen therapy, mechanical ventilation, vascular filling, catecholamines, anticonvulsants, antibiotics, gastric lavage, activated charcoal, and antidotes, while all patients underwent psychiatric evaluation; 95.1% were referred to outpatient follow-up and 4.9% to inpatient psychiatric care. Overall, 41.5% required hospitalization, with a mean stay of 2.4 ± 1.6 days (range: 1-18), and 97.6% had favorable outcomes, except for one patient who developed ventilator-associated pneumonia complicated by septic shock.

**Table 2:** clinical signs observed at admission among patients with acute drug intoxication at Mahres Regional Hospital, Tunisia, July 2021 - December 2023 (N = 41)

Clinical signs	Effective	Frequency % (n=41)
**Neurological signs**		
GCS 13-15	36	87.8
GCS 8-12	3	7.3
GCS < 8	2	4.9
Agitation	4	9.8
Myoclonus	5	12.2
Hypertonia	7	17.1
Hypotonia	3	7.3
Sharp reflexes	5	12.2
Reflexes abolished	6	14.6
Miosis	7	17.1
Mydriasis	2	4.9
Convulsions	2	4.9
Unrest	4	9.8
Dizziness	5	12.2
Visual blur	2	4.9
Headache	11	26.8
Hallucinations	13	31.7
Abnormal movements	3	7.3
Extrapyramidal symptoms	3	7.3
**Respiratory signs**		
Dyspnoea	2	4.9
Signs of struggle	2	4.9
Pulsed saturation< 92%	1	2.4
**Cardiovascular Signs**		
Tachycardia (Heart Rate>100 bpm)	7	17.1
Bradycardia (Heart Rate< 50 bpm)	5	12.2
Hypotension≤ 90 mm Hg	3	7.3
Hypertension≥ 140mm Hg	1	2.4
State of shock	2	4.9
**Digestive signs**		
Abdominal pain outside of epigastralgia	27	65.9
Epigastralgia	8	19.5
Nausea	16	39
Vomiting	32	78.1
Diarrhea	1	2.4

### Predictive factors of hospitalization

Univariate analysis identified several factors associated with hospitalization. Hospitalized patients more frequently had toxic habits and a history of psychiatric follow-up (Table 3) and were more likely to have ingested paracetamol, oral antidiabetics, anxiolytics, and antihypertensive drugs (Table 4). Clinically, they presented with lower heart rates and more frequent neurological symptoms, including headache, miosis, and seizures, as well as gastrointestinal symptoms such as epigastralgia and nausea. Laboratory parameters at admission did not differ significantly, whereas electrocardiographic abnormalities, specifically sinus bradycardia and conduction disorders, were more common (Table 5). Multivariate logistic regression identified independent predictors of hospitalization, including multi-drug intoxication, attainment of the toxic dose, paracetamol, oral antidiabetic and anxiolytic poisoning, miosis, seizures, sinus bradycardia, and conduction disorders. Only statistically significant predictors are presented in the text and in Table 6.

**Table 3:** selected demographic characteristics associated with hospitalization among patients with acute drug intoxication, Mahres Regional Hospital, Tunisia, July 2021 - December 2023 (N = 41)

Parameters	H+ Group (Effective=17)	H- Group (Effective =24)	P
Toxic Habits (N (%))	3 (17.6 %)	2 (8.3 %)	0.04
History of follow-up in psychiatry (N (%))	4 (23.5 %)	4 (16.7%)	0.01

**Table 4:** selected pharmacological agents associated with hospitalization among patients with acute drug intoxication, Mahres Regional Hospital, Tunisia, July 2021 - December 2023 (N = 41)

Pharmacological class	H+ Group (Effective = 17)	H-Group (Effective = 24)	P
Paracetamol	15 (88.2%)	12 (50 %)	0.02
PPI	5 (35.7%)	14 (58.3%)	0.07
NSAIDs	6 (35.3%)	11 (45.8 %)	0.5
Oral antidiabetic	3 (17.6%)	1 (4.2%)	0.04
Antihypertensive	11 (64.7%)	3 (12.5%)	0.002
Antibiotics	3 (17.6%)	8 (33.3 %)	0.8
Antidepressant	6 (35.3%)	4 (16.7 %)	0.8
Anxiolytic	12 (70.6 %)	4 (16.7 %)	0.01
Neuroleptic	2 (11.8 %)	3 (12.5 %)	0.6
Anti-vertigo	1 (5.9%)	0 (0%)	0.2

**Table 5:** selected clinical and electrocardiographic characteristics associated with hospitalization among patients with acute drug intoxication, Mahres Regional Hospital, Tunisia, July 2021 - December 2023 (N = 41)

	H+ Group (Effective=17)	H- Group (Effective =24)	P
Pulse (beat/min ± SD)	67.4 ± 16.2	85 ± 11.1	0.03
Miosis (N(%))	4 (23.5 %)	3 (12.5 %)	0.001
Seizures (N(%))	2 (11.8 %)	0	0.03
Headache (N(%))	9 (52.9 %)	2 (8.3 %)	0.01
Nausea	9 (52.9 %)	7 (29.2 %)	0.02
Sinus bradycardia (N (%))	4 (23.5 %)	1 (4.2%)	0.04
Conduction disorders (N (%))	3 (17.6 %)	0	0.02

**Table 6:** independent predictors of hospitalization among patients with acute drug intoxication admitted to the Emergency Department of Mahres Regional Hospital, Tunisia, July 2021 - December 2023 (N = 41)

Factors	OR	95% CI		P
		Min	Max	
Multi-drug intoxication	3.3	2.6	0.031	6.7
Reaching the toxic dose	1.7	1.1	0.04	3.5
Paracetamol poisoning	1.4	1.2	0.02	2.7
Oral antidiabetic drug poisoning	1.3	1	0.01	3.6
Anxiolytic poisoning	1 .9	0.8	0.02	3.2
Miosis	1.2	1.2	0.043	4.2
Convulsions	1.3	0.8	0.001	2.1
Headache	1.3	1	0.007	2.8
Sinus bradycardia	1.3	0.7	0.031	2.7
Conduction disorders	1.8	1.4	0.02	4.2

## Discussion

### Presentation of acute drug poisoning

The emergency diagnosis of acute drug intoxication relies primarily on a careful correlation between the suspected ingested dose and the patient´s presenting signs and symptoms. In medical toxicology, the diagnosis is largely guided by a thorough patient history and systematic clinical assessment. Clinical examination must be rigorous, structured, and dynamic, allowing continuous monitoring of the patient´s evolving condition. Essential components include the evaluation of vital signs, neurological status, and gastrointestinal, cardiovascular, and respiratory systems. An electrocardiogram (ECG) is recommended systematically when the poisoning is suspected to be severe or when complications are anticipated, as it can reveal conduction disorders, arrhythmias, or other life-threatening changes. Similarly, basic laboratory tests-such as serum electrolytes, creatinine, glucose, calcium, complete blood count, liver function tests, and arterial blood gases-are prioritized over toxicological screens for immediate clinical decision-making [2]. The prognosis depends on several factors, including the identity and dose of the toxicant, its formulation (immediate or sustained release), the patient´s comorbidities, the interval between exposure and treatment, the potential delayed onset of symptoms due to metabolic activation, and the presence of early complications [2]. Overdose situations can alter drug pharmacokinetics, resulting in rapid and unexpected elevations in plasma and tissue drug concentrations, which may worsen toxicity.

The concept of “toxidromes” was first introduced by Mofenson and Greensher in 1974 [3], is crucial in clinical toxicology. A toxidrome is a specific constellation of clinical signs, symptoms, laboratory abnormalities, and sometimes ECG changes, which collectively suggest a particular class of toxic agents. Recognizing toxidromes allows clinicians to identify poisoning when patient history is incomplete or unreliable, infer the mechanism of toxicity, and decide on the use of specific antidotes. A single toxic agent may produce multiple toxidromes, whereas multi-drug exposures or secondary complications can modify the classical clinical presentation. The syndromic approach is particularly valuable in challenging situations, such as patients who are unconscious, agitated, delirious, or in cardiorespiratory distress, where history-taking is impossible. By systematically identifying the relevant toxidrome, clinicians can streamline diagnosis, differentiate from non-toxic conditions, select appropriate antidotes, assess severity, and determine the necessary monitoring and therapeutic interventions [4]. In essence, a structured toxidromic approach enhances early recognition of life-threatening poisonings, improves patient triage, and guides targeted interventions, thereby optimizing outcomes in acute drug intoxication.

### Predictive factors for hospitalization

In our study, 61.2% of patients with acute drug poisoning required hospitalization. The decision to admit a patient and the duration of hospitalization vary according to patient and toxicant characteristics, and are further complicated by the increasing frequency of multiple-drug ingestions. Hachelaf *et al*. [5] reported that factors strongly associated with prolonged monitoring included the threshold ingested dose, multiple-drug poisoning, male sex, patient age, and the absence of activated charcoal administration, while the use of antidotes influenced prognosis without reducing hospital stay. Similarly, Liisanantti *et al*. [6] identified renal failure, respiratory failure, and thrombocytopenia at emergency department admission as predictors of prolonged hospitalization.

In our cohort, independent predictors of hospitalization included multiple-drug poisoning (OR = 3.3; 95% CI [2.6-6.7]; p = 0.031), reaching the toxic dose (OR = 1.7; 95% CI [1.1-3.5]; p = 0.04), paracetamol poisoning (OR = 1.4; 95% CI [1.2-2.7]; p = 0.02), oral antidiabetic poisoning (OR = 1.1; 95% CI [1.1-2.8]; p = 0.01), anxiolytic poisoning (OR = 1.9; 95% CI [0.8-3.2]; p = 0.02), miosis (OR = 1.2; 95% CI [1.2-4.2]; p = 0.043), seizures (OR = 1.3; 95% CI [0.8-2.1]; p = 0.001), sinus bradycardia (OR = 1.3; 95% CI [0.7-2.7]; p = 0.031), and conduction disorders (OR = 1.8; 95% CI [1.4-4.2]; p = 0.02). These findings highlight that predictors of hospitalization are highly dependent on the specific toxicant, its expected effects, and the objectives of each study.

The assessment of acute poisoning severity should rely on simple, rapidly available indicators in the emergency department. Decision-making criteria should be as objective as possible in order to harmonize practice between clinicians and centers. Patients' subjective complaints and estimated ingested doses should be supplemented-or preferably replaced-by measurable clinical parameters and readily accessible paraclinical data. Tools such as the Poison Severity Score (PSS) [7] were developed based on expert consensus, considering only functional or physical clinical signs while excluding estimated ingested doses or blood concentrations [8]. The PSS is easy to use and has been evaluated for nine toxicant categories in a multicenter study, although it is based on retrospective assessment and can only be applied after overall clinical evolution. The Toxscore [9] is more specifically adapted to toxicology, incorporating 30 objective variables derived from clinical and paraclinical assessments (including laboratory tests, chest radiography, and ECG), weighted from 0 to 4 according to the degree of abnormality. In addition, the Glasgow Coma Scale (GCS) was replaced in this system by a neuromuscular activity assessment scale, with neonatal and pediatric versions available.

Expert recommendations from the French Society of Intensive Care (SRLF) emphasize that ICU admission in poisoned patients should take into account the toxicant´s characteristics, estimated ingested dose, co-ingestions (with additive or synergistic effects), formulation type (immediate vs. sustained release), patient age and comorbidities, time elapsed between ingestion and management, delayed symptom onset related to toxicant metabolism, as well as the occurrence of complications [10]. They also note that commonly used physiological scores (IGS II, APACHE, Edinburgh scale, Reaction Level Scale [RLS]) are not suitable for establishing individual prognosis or for guiding clinical decisions in poisoned patients. Neither the Toxscore nor the PSS has yet been fully validated in toxicology, underscoring the need for objective and rapidly available parameters to guide hospitalization decisions.

## Conclusion

All of these results underline the major number of potentially preventable hospitalizations. A specific guideline for poisoning should be drawn up to standardise emergency procedures. Psychiatric care is mandatory to avoid recurrence.

### What is known about this topic

- Acute drug poisoning is a common cause of emergency department admissions;
- Certain drugs carry a high toxic risk in cases of overdose;
- Hospitalization criteria vary depending on clinical context.

### What this study adds

- This study identifies specific clinical and pharmacological predictors of hospitalization in patients with acute drug intoxication in Tunisia;
- It provides locally relevant evidence to support emergency physicians in making timely and objective decisions regarding hospital admission;
- It highlights the importance of simple, readily available clinical and ECG parameters in improving the early triage and management of acute drug intoxication.
